# State variation in opioid treatment policies and opioid-related hospital readmissions

**DOI:** 10.1186/s12913-018-3703-8

**Published:** 2018-12-17

**Authors:** Janice Blanchard, Audrey J. Weiss, Marguerite L. Barrett, Kimberly W. McDermott, Kevin C. Heslin

**Affiliations:** 1 RAND Corporation, 1200 Hayes Street, Arlington, VA 22202 USA; 2 George Washington University, 2120 L Street NW, Suite 450, Washington, DC 20037 USA; 3 IBM Watson Health, 5425 Hollister Avenue, Suite 140, Santa Barbara, CA 93111 USA; 4 ML Barrett, Inc., 13943 Boquita Drive, Del Mar, CA 92014 USA; 5 Agency for Healthcare Research and Quality, 5600 Fishers Lane, Rockville, MD 20857 USA

**Keywords:** Opioid abuse, Opioid readmissions, State opioid treatment policies, Medication-assisted treatment

## Abstract

**Background:**

State policy approaches designed to provide opioid treatment options have received significant attention in addressing the opioid epidemic in the United States. In particular, expanded availability of naloxone to reverse overdose, Good Samaritan laws intended to protect individuals who attempt to provide or obtain emergency services for someone experiencing an opioid overdose, and expanded coverage of medication-assisted treatment (MAT) for individuals with opioid abuse or dependence may help curtail hospital readmissions from opioids. The objective of this retrospective cohort study was to evaluate the association between the presence of state opioid treatment policies—naloxone standing orders, Good Samaritan laws, and Medicaid medication-assisted treatment (MAT) coverage—and opioid-related hospital readmissions.

**Methods:**

We used 2013–2015 hospital inpatient discharge data from 13 states from the Agency for Healthcare Research and Quality Healthcare Cost and Utilization Project. We examined the relationship between state opioid treatment policies and 90-day opioid-related readmissions after a stay involving an opioid diagnosis.

**Results:**

Our sample included 383,334 opioid-related index hospitalizations. Patients treated in states with naloxone standing-order policies at the time of the index stay had higher adjusted odds of an opioid-related readmission than did those treated in states without such policies; however, this relationship was not present in states with Good Samaritan laws. Medicaid methadone coverage was associated with higher odds of readmission among all insurance groups except Medicaid. Medicaid MAT coverage generosity was associated with higher odds of readmission among the Medicaid group but lower odds of readmission among the Medicare and privately insured groups. More comprehensive Medicaid coverage of substance use disorder treatment and a greater number of opioid treatment programs were associated with lower odds of readmission.

**Conclusions:**

Differences in index hospitalization rates suggest that states with opioid treatment policies had a higher level of need for opioid-related intervention, which also may account for higher rates of readmission. More research is needed to understand how these policies can be most effective in influencing acute care use.

## Background

The opioid epidemic in the United States has escalated in recent years, subsequently affecting the U.S. health care system. From 2002 to 2015, there was a 2.8-fold increase in the number of opioid-related deaths [1]. From 2005 to 2014, hospitalizations involving opioids increased by 64% [2]. Three state policy approaches designed to provide opioid treatment options have received significant attention: (1) expanded availability of naloxone to reverse overdose, (2) Good Samaritan laws intended to protect individuals who attempt to provide or obtain emergency services for someone experiencing an opioid overdose, and (3) expanded coverage of medication-assisted treatment (MAT) for individuals with opioid abuse or dependence.

Naloxone can rapidly reverse the potentially life-threatening effects of opioids. It has low rates of adverse events and can be administered by laypersons [3]. Some states allow *standing orders* that make naloxone directly available without an individualized provider prescription from sites such as community agencies or pharmacies [4–6]. As of June 2016, 47 states and the District of Columbia had passed some type of law increasing naloxone availability, including laws that allow standing orders at pharmacies [5].

Good Samaritan opioid-related legislation provides individuals who call 911 for an opioid overdose with immunity from arrest, charge, or prosecution for certain drug-related violations [7]. As of January 2017, 34 states and the District of Columbia had Good Samaritan laws in place [4, 5].

The Food and Drug Administration (FDA) has approved three principal drugs as part of MAT to help individuals reduce the use of opioids—methadone, buprenorphine (with or without naloxone), and naltrexone (injectable and pill form). The American Society of Addiction Medicine (ASAM) guidelines recommend coverage of all three drugs [8, 9]. Medicaid, the largest payer of substance use disorder (SUD) services, covers buprenorphine/naloxone and at least one form of naltrexone in most states; however, many states impose some restrictions such as prior authorization, dosing limits, or SUD counseling prior to prescribing [8, 9]. Many state Medicaid agencies do not cover methadone, which must be administered in an opioid treatment program (OTP) that meets federal counseling requirements [4, 9].

Naloxone standing orders, Good Samaritan laws, and expanded MAT coverage may affect hospitalizations related to opioid-related adverse events, overdoses, and deaths, while also encouraging individuals with opioid use disorders to seek treatment [4, 10]. However, research evaluating the association between acute care use and these three state policies is limited, with prior studies focused only on a subset of these policies, such as coverage of methadone maintenance, or restricted to single state evaluations [10, 11].

The objective of this retrospective cohort study was to evaluate the relationship between these three opioid treatment policies and the risk of opioid-related readmission. We used 2013–2015 hospital discharge data from 13 states with differing treatment policies to estimate the odds of a subsequent opioid-related readmission within 90 days following discharge. We hypothesized that naloxone standing orders would be associated with lower odds of readmission because they would allow individuals with an opioid overdose to obtain treatment outside of the acute care setting, thereby reducing the need for hospitalization [4]. Conversely, we hypothesized that Good Samaritan laws may increase acute care hospital use because they would cause more individuals to alert the emergency medical system of an opioid overdose [7]. We further hypothesized that Medicaid MAT coverage would be associated with lower odds of readmission for an opioid-related diagnosis in the Medicaid population because it would increase the availability of treatment [11]. Medicaid coverage may have spillover effects because providers and facilities that treat Medicaid populations also may treat other insured populations [12] and may be more aware of MAT in states with more generous Medicaid coverage. For this reason, we hypothesized that Medicaid MAT coverage also would be associated with lower odds of readmission for patients with non-Medicaid coverage.

## Methods

### Data source

We used inpatient discharge data from nonfederal community hospitals in Arkansas, California, Florida, Georgia, Iowa, Maryland, Massachusetts, Nebraska, Nevada, New York, Tennessee, Vermont, and Wisconsin from the Agency for Healthcare Research and Quality (AHRQ) Healthcare Cost and Utilization Project (HCUP) [13]. We included states with encrypted patient linkage numbers to link records from the 2013, 2014, and 2015 (quarters 1 through 3) HCUP State Inpatient Databases (SID) [14] during the study period.^1^ We also included only those states with data indicating whether diagnoses were present on admission (POA) to exclude index stays (i.e., initial hospitalizations) involving an opioid diagnosis that may have occurred solely because of hospital-related factors, such as iatrogenic complications of opioid use.

We obtained state-level data about the status and specific implementation dates of naloxone standing orders and Good Samaritan laws from The Policy Surveillance Program: A Law Atlas Project [6, 7]. For state Medicaid MAT policies, we could determine the status of these policies for the 2013–2014 period, but specific implementation dates were unavailable. Our key sources of MAT Medicaid policy information included ASAM state reports [4, 15–27], a 2016 article by Grogan and colleagues about state Medicaid MAT benefits [9], and personal communication with the Grogan article authors. When data from these sources were incomplete, we used several supplementary sources including two state Medicaid Preferred Drug Lists [28, 29], contacts at five state Medicaid agencies, and a Kaiser Family Foundation (KFF) report on rehabilitative services [30].

We gathered information about presence of hospital detoxification and psychiatry units from the American Hospital Association [31]. For each data year, we obtained the state population capacity of facilities to treat SUDs (facilities offering care for SUDs including outpatient, residential, and inpatient hospital treatment for all payer categories), number of OTPs, and number of providers newly certified to administer buprenorphine/naloxone from the Substance Abuse and Mental Health Services Administration (SAMHSA) National Survey of Substance Abuse Treatment Services [32–34] and the SAMHSA Number of Drug Addiction Treatment Act (DATA)-Waived Practitioners Newly Certified Per Year tracker [35]. We also used Grogan and colleagues (2016) [9] to obtain data about state Medicaid coverage of ASAM-recommended SUD treatment levels from 2013 through 2014. Finally, we obtained state rates of opioid overdose deaths for each data year from the Kaiser Family Foundation State Health Facts database [36].

### Study population

The study population comprised a retrospective longitudinal sample of patients aged 18 years and older with an opioid-related index hospitalization between April 2013 and June 2015 and no preceding opioid-related hospitalization within 90 days.^2^ Opioid-related stays were identified by any-listed *International Classification of Diseases, Ninth Revision, Clinical Modification (ICD-9-CM)* diagnosis codes that were present on admission for opioid abuse or dependence alone or in combination with other drugs (304.00–304.02, 304.70–304.72, 305.50–305.52) or for poisoning by opium, methadone, heroin, opiates and related narcotics, or opiate antagonists (965.00–965.02, 965.09, 970.1). We also included external cause of injury codes (E codes) for accidental poisoning by opium, methadone, heroin, and opiates and related narcotics (E850.0–E850.2) and adverse effects of heroin, methadone, opiates and other narcotics, and opiate antagonists (E935.0–E935.2, E940.1). We included any-listed opioid diagnoses for our index stays to capture the potential population of individuals who could be affected by naloxone, Good Samaritan, and MAT coverage policies. We excluded index hospitalizations in which the patient died or was transferred into or out of the hospital.

### Outcome variable: Readmissions

The outcome variable was a readmission within a 90-day period with an opioid-related principal diagnosis or an opioid-related accidental poisoning or adverse effect diagnosis (E code). Consistent with other studies, we selected 90 days as the follow-up period because it would be sufficient time for patients discharged from the hospital to access potential outpatient rehabilitation services [37, 38]. This limited readmissions to hospitalizations that potentially would be most affected by our state policies of interest and excluded hospitalizations in which opioid-related diagnoses were only a secondary concern.

### Key independent variables: State policies for opioid treatment

The key independent variables focused on the three state policies: (1) naloxone standing orders, (2) Good Samaritan laws, and (3) Medicaid MAT coverage and generosity.

The first key independent variable indicated whether a state had a naloxone standing order that allowed pharmacies to dispense naloxone without an individual provider prescription. The second indicated whether a state had a Good Samaritan law granting immunity to users from arrest, charge, or prosecution for possession of drugs or drug paraphernalia. For these first two independent variables, we classified an index stay as having a naloxone standing order or Good Samaritan law if the date of implementation was before or on the date of the index stay [6, 7].

The final two key independent variables represented Medicaid MAT coverage and generosity. Because the states in our sample had little variation in naltrexone and buprenorphine/naloxone coverage, we focused on two components of MAT coverage: whether a state had any coverage of methadone for Medicaid enrollees and whether a state had more or less generous coverage of buprenorphine/naloxone or naltrexone for Medicaid enrollees. Generosity of coverage of buprenorphine/naloxone or naltrexone was a composite variable based on the following four measures: (1) prior authorization requirement for buprenorphine/naloxone, (2) prior authorization requirement for injectable naltrexone, (3) buprenorphine/naloxone dosing limits (either limitation on total days coverage or maximum dosage restrictions of less than 24 mg/day), and (4) requirement for SUD counseling prior to treatment with buprenorphine/naloxone or naltrexone. If a state lacked restrictions on at least two of these four measures, it was categorized as more (vs. less) generous. As noted above, the Medicaid methadone coverage and MAT coverage generosity measures were based on data collected from a combination of sources. Because these data sources did not provide exact dates of implementation, we counted a state as having coverage in place during the years in which our sources collected the data (2013–2014) [4, 9, 15–27].

### Covariates

The analysis included covariates for patient-level factors, hospital stay and hospital characteristics, and state-level factors—all measured at the time of the index stay—that could have influenced the outcome of 90-day readmission. Patient-level factors included sociodemographic characteristics: age (continuous variable), sex, race/ethnicity (White, Black, Hispanic, other, missing), expected primary payer (Medicare, Medicaid, private insurance, uninsured/self-pay, other), community-level income based on the state-defined quartile for median household income of the ZIP Code of the patient’s residence, and urban/rural residency. To examine severity of illness, we identified whether the primary reason for the admission (principal diagnosis) was an opioid-related diagnosis or a non-opioid-related diagnosis, whether the patient was admitted with an opioid use disorder diagnosis (304.00–304.02, 304.70–304.72, 305.50–305.52) or a poisoning/adverse effect diagnosis (965.00–965.02, 965.09, 970.1, E850.0–E850.2, E935.0–E935.2, E940.1), and whether the patient had a continuous opioid use disorder (304.01, 304.71, 305.51). We used the HCUP Clinical Classifications Software (CCS) diagnosis categories [39] to define any-listed co-occurring mental health conditions (CCS 650–652, 655–659, 662, 670) or alcohol-related conditions (CCS 660). We used the Elixhauser Comorbidity Software [40] to create dichotomous variables indicating whether the stay involved a specific co-occurring physical (medical) condition(s); the count of the co-occurring physical condition(s) also was included.

Hospital stay characteristics included whether patients received any treatment for drug rehabilitation or detoxification during the index stay (ICD-9-CM procedure codes 94.64–94.69) and the length of the index stay. Because SUD treatment varies across hospitals, we included covariates for characteristics related to the hospital in which the index admission occurred: percentage of hospital discharges among patients with opioid-related conditions and whether the hospital had a SUD detoxification or psychiatric acute care unit.

State-level factors included the following measures of capacity for opioid treatment that were based on the year of the index stay: Medicaid coverage of all four levels of ASAM-recommended treatment services (outpatient, intensive outpatient, inpatient, intensive inpatient) during the 2013–2014 period [9]; newly certified provider capacity for buprenorphine/naloxone therapy, defined as the number of newly eligible DATA-waived practitioners approved to provide buprenorphine/naloxone treatment in a state per 100,000 population [35]; the number of OTPs per 100,000 population; and the number of SUD treatment facility beds per 100,000 population [32–34]. We included covariates for year of index stay, source of admission (emergency department vs. direct admission), and state overdose death rates to denote state-wide severity of opioid use. Because we hypothesized that the state MAT policies would have a direct effect on the Medicaid population and spillover effects on other insurance populations, we included interaction terms for Medicaid MAT coverage and generosity with each payer group.

### Analysis

We first conducted bivariate analysis to examine characteristics of our sample as well as the association between our key independent (policy) variables and the outcome variable, opioid-related readmission within 90 days after discharge. Next, we conducted multivariate logistic regression analysis to estimate the association between our key independent variables and an opioid-related readmission, taking into account the patient-, hospital- and state-level factors described above.

All data were analyzed using SAS version 9.4. The HCUP databases are consistent with the definition of limited data sets under the Health Insurance Portability and Accountability Act Privacy Rule and contain no direct patient identifiers. The AHRQ Human Research Protections Program has determined that research using HCUP data has exempt status.

## Results

### Bivariate analysis

During the study period, there were 383,334 index hospitalizations involving opioid-related diagnoses across the 13 sample states. Table 1 shows characteristics of the sample of index hospital stays by each policy category. Patients treated in states with naloxone standing orders, with Good Samaritan laws, and that offered Medicaid coverage of methadone and more generous Medicaid coverage of MAT had higher rates of diagnoses of continuous opioid abuse and dependence on index stays. These patients also were more likely to be covered by Medicaid and less likely to be White compared with patients treated in states without such policies. States that offered Medicaid coverage of methadone and more generous Medicaid MAT coverage had more index stays from patients in the lower income quartiles and more stays from patients in urban areas. States with Medicaid methadone coverage had a lower capacity of newly certified providers offering office-based buprenorphine/naloxone therapy but a higher capacity of OTPs and SUD treatment facilities compared with states that did not offer such coverage. States that offered more generous MAT coverage had higher capacity of newly-certified office-based buprenorphine/naloxone providers, OTPs, and SUD facilities compared with states that offered less generous coverage.

**Table 1:** Characteristics of opioid-related index hospitalizations by state policy category

Characteristic	Naloxone Standing Orders		Good Samaritan Laws		Methadone Coverage by Medicaid		MAT Coverage Generosity by Medicaid^a^	
	Yes	No	Yes	No	Yes	No	More Generous	Less Generous
Number of stays	145,262	238,072	307,369	75,965	353,335	29,999	237,221	146,113
Patient Sociodemographic Characteristics								
Age (mean)	47.7**	46.3	47.0**	46.0	46.9**	45.8	45.0**	49.7
Female (%)	48.1**	49.4	47.7**	53.7	48.2**	57.4	46.5**	52.8
Race/ethnicity (%)								
White	68.6 **	70.1	68.6**	73.2	68.2**	85.5	67.9**	72.2
Black	12.4**	14.2	12.8**	16.5	14.2**	5.9	15.3**	10.7
Hispanic	13.0**	9.3	12.7**	2.7	11.4 **	2.4	10.0**	12.0
Other	5.5**	4.1	5.3**	1.9	4.9**	0.6	5.3**	3.5
Missing	0.5**	2.3	0.6**	5.7	1.3**	5.5	1.6	1.6
Expected primary payer (%)								
Medicare	31.5**	30.1	30.5**	31.4	30.2**	36.1	27.0**	36.5
Medicaid	42.4**	34.7	38.9**	32.5	38.6**	26.1	42.3**	30.0
Private insurance	18.0**	17.5	17.7*	17.4	17.5**	17.5	16.6**	19.3
Uninsured/self-pay	5.1**	13.2	8.8**	15.5	9.7**	14.7	10.9**	8.8
Other	3.0**	4.4	4.1**	2.8	4.0**	2.0	3.1**	5.1
Median community-level income (%)								
Quartile 1 (lowest)	31.7**	34.9	32.4**	38.9	34.0**	29.9	35.9**	30.1
Quartile 2	24.6**	23.8	23.9**	25.0	23.7**	28.8	22.6**	26.5
Quartile 3	21.1*	20.8	21.1**	20.2	20.6**	24.2	19.9**	22.5
Quartile 4 (highest)	17.6**	16.4	17.5**	14.3	16.9**	15.7	16.5**	17.4
Missing	5.0**	4.1	5.2**	1.6	4.7**	1.4	5.1**	3.4
Patient location (%)								
Urban	89.7**	90.7	92.4**	82.0	92.0**	70.9	93.1**	85.8
Rural	8.9**	8.2	6.2**	17.7	6.7**	28.9	5.9**	12.6
Patient Clinical Factors								
Opioid-related diagnosis (%)								
Principal	13.3**	13.8	13.7*	13.4	13.3**	17.0	14.4**	12.2
Secondary only	86.7**	86.2	86.3*	86.6	86.7**	83.0	85.6**	87.8
Any opioid abuse or dependence (%)	83.0	82.9	83.6**	80.0	83.4**	77.4	85.4**	78.8
Continuous opioid abuse or dependence (%)	29.9*	29.6	30.8**	25.2	30.4**	21.4	32.6**	24.9
Mental health co-occurring diagnosis (%)	54.1**	55.7	54.2**	58.9	54.6**	61.3	55.7**	54.2
Alcohol abuse co-occurring diagnosis (%)	19.5	19.5	19.7**	18.6	19.9**	14.5	21.3**	16.5
Number of co-occurring physical conditions (mean)^b^	2.1**	2.0	2.0**	2.1	2.0**	1.9	1.8**	2.3
Initial Hospital Stay Characteristics								
Hospital stay began in ED (mean %)	75.0**	76.7	77.4**	70.9	77.2 **	63.5	76.4**	75.5
Length of index stay (mean days)	5.3**	5.1	5.3**	4.6	5.2**	4.5	5.2**	5.1
Receipt of rehabilitation during index stay (%)	16.9**	16.1	16.9**	14.7	16.5	16.3	21.2**	8.8
Hospital Characteristics^c^								
Percentage of annual opioid-related discharges in index year (mean)	3.9	3.9	3.9	3.9	3.9**	3.2	4.7**	2.5
Alcohol/substance use disorder (SUD) detoxification unit (%)								
No	56.8**	59.3	57.5**	61.8	58.0**	62.1	53.0**	67.0
Yes	26.7 **	24.3	25.9**	22.3	25.7**	19.4	32.8**	12.8
Not reported	16.6	16.4	16.6**	15.9	16.3**	18.5	14.2**	20.1
Psychiatric acute care unit (%)								
No	35.9**	30.9	32.9*	32.5*	32.7**	34.7	25.7**	44.4
Yes	47.5**	52.8	50.6**	51.6	51.1**	46.8	60.1**	35.7
Not reported	16.6*	16.3	16.5**	15.9	16.2**	18.5	14.2**	19.9
State Factors								
Medicaid coverage of ASAM recommended treatment services (mean)	87.1**	58.7	72.0**	59.1	69.2**	72.6	65.0**	76.6
Newly certified provider capacity for buprenorphine/naloxone per 100,000 state population during year of index stay (mean)	86.2**	76.4	79.9**	80.9	78.8**	96.1	95.6**	55.0
Number of opioid treatment programs per 100,000 state population during year of index stay (mean)	0.6*	0.6	0.6**	0.5	0.6**	0.1	0.7**	0.3
SUD treatment capacity per 100,000 state population during year of index stay (mean)	43.4**	42.2	45.2**	32.4	43.7**	30.5	48.6**	33.0
Opioid overdose death rate during year of index stay (mean)	9.1**	9.3	8.7**	11.4	9.1**	11.0	10.7**	6.9

*ASAM* American Society of Addiction Medicine, *MAT* medication-assisted treatment, *OTP* opioid treatment program, *SUD* substance use disorder

*p < 0.05; **p < 0.001

^a^This is a composite variable of preauthorization requirements for buprenorphine/naloxone, preauthorization for naltrexone, dosing limits for buprenorphine/naltrexone, and SUD counseling requirements

^b^Co-occurring conditions were formulated from 25 Elixhauser comorbidities (excluding mental health and SUD variables, which are reported as separate variables)

^c^Hospital characteristics describe the hospital at which the index visit occurred

Bivariate analysis showed that states with naloxone standing orders, Good Samaritan laws, Medicaid coverage of methadone, and more generous Medicaid coverage of MAT had a higher percentage of opioid-related readmissions within 90 days compared with states without these policies (Table 2).

**Table 2:** Bivariate results: unadjusted association of 90-day readmission and state policy

State Policy	% Readmitted Within 90 Days	95% CI
Naloxone standing orders		
Yes	3.2*	3.1–3.2
No	2.8	2.8–2.9
Good Samaritan laws		
Yes	3.2*	3.2–3.3
No	1.7	1.6–1.8
Methadone coverage by Medicaid		
Yes	3.0*	3.0–3.1
No	1.8	1.6–1.9
MAT coverage generosity by Medicaid^a^		
More generous	3.5*	3.5–3.6
Less generous	2.0	1.9–2.1

*CI* confidence interval, *MAT*, medication-assisted treatment

*p < 0.001

^a^This is a composite variable of preauthorization requirements for buprenorphine/naloxone, preauthorization for naltrexone, dosing limits for buprenorphine/naltrexone, and substance use disorder counseling requirements

### Multivariate analysis

Table 3 shows the results of our multivariate analysis. Patients in states with naloxone standing orders had higher odds of an opioid-related readmission (OR = 1.14, 95% CI = 1.07–1.20) compared with patients in states without naloxone standing orders. There was no significant relationship between Good Samaritan laws and opioid-related readmission.

**Table 3:** Multivariate results: adjusted association of 90-day readmission and state policies^a^

Variable	90-Day Readmission	
	OR	95% CI
Key Independent Variables		
Naloxone standing orders	1.14***	1.07–1.20
Good Samaritan laws	1.07	0.97–1.19
Methadone coverage by Medicaid, by payer (reference: without methadone coverage)^b^		
Medicaid	1.23	0.97–1.56
Medicare	1.40**	1.15–1.72
Private insurance	1.87***	1.44–2.41
Uninsured	1.57*	1.06–2.33
Other insurance/missing	0.99	0.56–1.72
MAT coverage generosity by Medicaid, by payer (reference: less generous coverage)^c^		
Medicaid	1.28**	1.11–1.48
Medicare	0.80**	0.70–0.91
Private insurance	0.74***	0.63–0.85
Uninsured	1.20	0.95–1.51
Other insurance/missing	1.14	0.86–1.50
Covariates		
Patient Sociodemographic Characteristics		
Age	0.99***	0.99–0.99
Female	0.86***	0.83–0.90
Race/ethnicity (reference: White)		
Black	0.97	0.91–1.03
Hispanic	1.05	0.98–1.11
Other	0.97	0.89–1.05
Missing	0.78*	0.63–0.97
Median community-level income (reference: Quartile 4, highest)		
Quartile 1 (lowest)	0.87***	0.82–0.92
Quartile 2	0.84***	0.79–0.89
Quartile 3	0.87***	0.82–0.92
Missing	0.95	0.86–1.05
Patient location: rural (reference: urban)	0.84***	0.78–0.92
Patient Clinical Factors		
Opioid-related diagnosis: principal (reference: secondary diagnosis only)	1.88***	1.79–1.98
Any opioid abuse or dependence	0.83***	0.76–0.90
Continuous opioid abuse or dependence	1.16***	1.10–1.21
Mental health co-occurring diagnosis	1.09**	1.03–1.15
Alcohol abuse co-occurring diagnosis	1.01	0.96–1.06
Number of co-occurring physical conditions^d^	1.29**	1.08–1.53
Initial Hospital Stay Characteristics		
Hospital stay began in emergency department	1.16***	1.11–1.22
Length of index stay	0.98***	0.98–0.99
Receipt of rehabilitation during index stay	1.96***	1.84–2.09
Hospital Characteristics		
Percentage of annual opioid-related discharges in index year	1.02***	1.02–1.03
Alcohol/ SUD detoxification unit (reference: no unit)		
Yes	1.27***	1.20–1.34
Not reported	0.90	0.33–2.43
Psychiatric acute care unit (reference: no unit)		
Yes	1.01	0.96–1.06
Not reported	1.23	0.45–3.33
State Factors		
Medicaid coverage of all four ASAM recommended services	0.74***	0.66–0.82
Newly certified provider capacity for buprenorphine/naloxone per 100,000 state population during year of index stay	1.00*	1.00–1.00
Number of opioid treatment programs per 100,000 state population during year of index stay	0.35***	0.28–0.44
SUD treatment capacity per 100,000 state population during year of index stay	1.04***	1.03–1.04
Opioid overdose death rate during year of index stay	1.02*	1.01–1.04

*ASAM* American Society of Addiction Medicine, *CI* confidence interval, *MAT* medication-assisted treatment, *OR* odds ratio, *SUD* substance use disorder

*p < 0.05; **p < 0.01; ***p < 0.001

^a^The model also controlled for co-occurring physical conditions and year of discharge, which are not listed in the table

^b^Odds ratios were calculated by using an interaction term for Medicaid methadone coverage and each of the payer groups

^c^This is a composite variable of preauthorization requirements for buprenorphine/naloxone, preauthorization for naltrexone, dosing limits for buprenorphine/naltrexone, and substance use disorder counseling requirements. Odds ratios were calculated by using an interaction term for Medicaid MAT generosity and each of the payer groups

^d^Co-occurring conditions were formulated from 25 Elixhauser comorbidities (excluding mental health substance abuse variables, which are reported as separate variables)

By insurance group, compared with states without Medicaid methadone coverage, the odds of readmission in states with Medicaid methadone coverage were higher among Medicare patients (OR = 1.40, 95% CI = 1.15–1.72), privately insured patients (OR = 1.87, 95% CI = 1.44–2.41), and uninsured patients (OR = 1.57, 95% CI = 1.06–2.33). Medicaid methadone coverage was not associated with readmission among Medicaid patients.

The odds of readmission in states with more generous Medicaid MAT coverage were lower among Medicare and privately insured patients (OR = 0.80, 95% CI = 0.70–0.91; OR = 0.74, 95% CI = 0.63–0.85, respectively) but higher among Medicaid patients (OR = 1.28, 95% CI = 1.11–1.48) compared with these same groups of patients in states with less generous Medicaid MAT coverage. Medicaid MAT coverage generosity was not associated with readmissions among uninsured patients.

Among the covariates in our model, patients treated in states where Medicaid covered all four ASAM-recommended treatment levels and states with more OTPs had lower odds of an opioid-related readmission (OR = 0.74, 95% CI = 0.66–0.82; OR = 0.35, 95% CI = 0.28–0.44, respectively) than did patients in states without these capacities. By contrast, patients in states with more substance use facility beds (which included residential and inpatient beds for all payers) and a higher opioid death rate had higher odds of readmission (OR = 1.04, 95% CI = 1.03–1.04; OR = 1.02, 95% CI = 1.01–1.04, respectively).

Other factors associated with higher odds of an opioid-related readmission included the following at index hospitalization: a continuous opioid-related diagnosis, an opioid principal diagnosis, more physical codiagnoses, a mental health codiagnosis, and receipt of inpatient detoxification or rehabilitation treatment. In addition, patients with index stays in hospitals with more opioid-related discharges or with dedicated SUD treatment units had higher odds of readmission. By contrast, patients who were female, from the lowest income quartile or rural areas, with an opioid use disorder diagnosis (vs. a poisoning or adverse event diagnosis), or with a longer index stay had lower odds of readmission.

## Discussion

In this study, patients with an opioid-related hospitalization in states with naloxone standing orders, more generous Medicaid methadone coverage (for the privately insured, Medicare, and uninsured groups), and more generous Medicaid MAT coverage (for the Medicaid group) had higher odds of an opioid-related readmission within 90 days. It is likely that policies that address opioid use disorders, such as the ones we explored, were implemented in states that had high need for opioid-related intervention. For example, states with more generous Medicaid MAT coverage had higher opioid death rates. Patients in states with these policies were more likely to be admitted for continuous opioid use and more likely to be covered by Medicaid than patients from states without these policies. Another possible explanation for our findings is that states that implemented these policies had more aggressive lobbying efforts to promote passage of such laws coupled with educational campaigns that resulted in an increase in patient awareness about the importance of acute treatment for opioids [41].

Contrary to our hypothesis, patients in states with naloxone standing orders were more likely to be readmitted for opioid use than were patients in states without these policies. Prior research has shown that naloxone may reduce overdose mortality [42]. For example, a Massachusetts study showed that communities that distributed nasal naloxone had a reduction in overdose deaths compared with those that did not [10]. One explanation for our study’s findings is that by preventing deaths outside of the hospital, naloxone access may allow individuals to survive the initial opioid overdose and get to the hospital to seek care.

We did not find any relationship between Good Samaritan laws and opioid-related readmissions. We may not have had sufficient time to see the effects of these laws, many of which were implemented recently [7]. Despite the increasing adoption of Good Samaritan laws, many individuals who are at the greatest risk of opioid overdose are unaware of their existence or scope. A 2015 survey in Rhode Island of young adults who reported nonmedical use of prescription opioids showed that fewer than half knew about the state’s Good Samaritan law, enacted in 2012 [43]. To be most effective, it is likely that these laws need to be accompanied by educational campaigns alerting potential beneficiaries of their existence.

This analysis showed associations of varying magnitude and significance, depending on insurance coverage, between opioid-related readmission and Medicaid coverage of methadone and Medicaid MAT coverage generosity. Methadone coverage did not have a statistically significant association with readmission in the Medicaid population. However, in all other payer populations, individuals treated in states with Medicaid methadone coverage had higher odds of readmission. In prior studies among the Medicaid population, methadone use has been associated with an increased likelihood of acute care use for overdoses from opioids [44–46]. When patients present to OTPs for methadone, they have access to providers who may identify the need for acute care for opioid-related diagnoses that may warrant medical intervention. Medicaid coverage of methadone may help provide funding to OTPs that allow patients, regardless of insurance status, to obtain such access to care [4, 12]. In 2003, after Oregon temporarily discontinued Medicaid coverage of methadone, providers reported decreases in staff support and services related to the loss of this funding [12].

Medicaid MAT coverage generosity was associated with lower odds of an opioid-related readmission among individuals covered by Medicare or private insurance but higher odds of readmission for those covered by Medicaid. The reason is not entirely clear. The Medicaid population historically has had high rates of readmission, particularly for SUDs [38]. Our finding may reflect lower access to resources needed for outpatient rehabilitation services and coordinated care after an acute care episode among individuals covered by Medicaid [38, 47]. Individuals covered by private insurance and Medicare in states with generous Medicaid MAT coverage may be seeing benefits to therapy not otherwise realized among individuals covered by Medicaid, suggesting some spillover effects to privately insured and Medicare beneficiaries (e.g., because of greater provider awareness of MAT), perhaps combined with better access to other outpatient support services.

The findings also demonstrated that outpatient treatment capacity, along with coverage, is an important factor associated with readmission, particularly among the Medicaid population. Many states face barriers to distribution of MAT that extend beyond coverage, including lack of available providers, which tends to be most severe for publicly funded facilities [48]. In this analysis, individuals in states with more OTP facilities that distribute methadone, and often other forms of MAT, had lower odds of readmission. In addition, individuals in states where Medicaid covered all four ASAM levels of SUD treatment had lower odds of readmission. Even if a state offers Medicaid coverage of MAT, individuals in states with low availability of the types of providers needed to deliver MAT likely would not benefit from these coverage policies. After Massachusetts expanded coverage of SUD services, use of the services remained essentially flat—possibly because of a lack of expansion of infrastructure or sufficient engagement of clients [49].

Our study had some limitations. We did not have information in our sample on individual patient treatment or on whether individuals received treatment outside of the acute care medical or surgical hospital setting. We also did not have information on events that occurred outside of the acute care setting, such as post-discharge deaths, that may have affected our outcomes. Although we had the exact dates of implementation for naloxone standing orders and Good Samaritan laws, we did not have the exact dates of implementation for Medicaid methadone or MAT coverage policies. For the latter, we relied on general implementation information for the range of years during our study’s data collection period. Another limitation is that the association between our outcome and our policy variables may have been related to unmeasured variables; in addition, there may have been unmeasured endogenous terms that may have similarly impacted both the outcome and predictor variables. In addition, the available policy information did not distinguish between Medicaid fee-for-service and managed care for most states in our sample. Medicaid managed care plans may have separate formularies from fee-for-service formularies that may involve medication restrictions and prior authorization and utilization review requirements. Our sources indicated, however, that states in our sample had similar SUD coverage requirements under both types of plans. Finally, since our analysis was limited to 13 states, our results may not be generalizable to other regions or the nation as a whole.

## Conclusions

Policies to address opioid use disorders such as naloxone standing orders, Good Samaritan laws, and MAT coverage are still in their nascent stages but have the potential to affect acute care utilization, including readmissions. The implementation of such policies in the states included in this study may have been motivated by higher rates of continuous opioid abuse and dependence in the population. Although some policies were associated with increased rates of readmissions, others were associated with a decrease or no change in readmission rates. We propose some explanations for our findings; however, more research is needed to understand how these policies can be most effective in influencing acute care utilization, including educating the public about the existence of these benefits and ensuring that states have adequate capacity to optimize their effectiveness.

## Abbreviations

AHRQ: Agency for Healthcare Research and Quality
ASAM: American Society of Addiction Medicine
CCS: Clinical Classifications Software
DATA: Drug Addiction Treatment Act
FDA: The Food and Drug Administration
HCUP: Healthcare Cost and Utilization Project
ICD-9-CM: *International Classification of Diseases, Ninth Revision, Clinical Modification*
MAT: Medication-assisted treatment
OTP: Opioid treatment program
POA: Present on admission
SID: State Inpatient Databases
SUD: Substance use disorder

## Footnotes

1 We did not include data for quarter 4 of 2015 because of the transition during this time from the ICD-9-CM to the ICD-10-CM/PCS coding system, which previous research suggests may have issues with compatibility [50].

2 We used hospital discharges in January through March 2013 only for assessment of the 90-day pre-index period and discharges from July through September 2015 only for assessment of the 90-day post-index readmission period.
